# Effect of renin-angiotensin-aldosterone system inhibitors on survival outcomes in cancer patients treated with immune checkpoint inhibitors: a systematic review and meta-analysis

**DOI:** 10.3389/fimmu.2023.1155104

**Published:** 2023-04-19

**Authors:** Jinhai Shen, Hui Hou, Bowen Liang, Xiao Guo, Li Chen, Yong Yang, Yun Wang

**Affiliations:** ^1^ Center for New Drug Safety Evaluation and Research, China Pharmaceutical University, Nanjing, Jiangsu, China; ^2^ State Key Laboratory of Natural Medicines, China Pharmaceutical University, Nanjing, Jiangsu, China; ^3^ Department of Pharmacology, Suzhou Institute for Drug Control, Suzhou, Jiangsu, China; ^4^ School of Pharmacy, Xuzhou Medical University, Xuzhou, Jiangsu, China

**Keywords:** immune checkpoint inhibitors, renin-angiotensin-aldosterone system inhibitors, cancer, survival, meta-analysis

## Abstract

**Background:**

Effect of renin-angiotensin-aldosterone system inhibitors (RAASIs) in combination with immune checkpoint inhibitors (ICIs) on prognoses in cancer patients remains controversial. This study systematically evaluated the effect of RAASIs on survival outcomes in cancer patients receiving ICIs treatment and provided an evidence-based reference for the rational use of RAASIs and ICIs combination therapy in clinical practice.

**Methods:**

Studies evaluating the prognosis of RAASIs-used versus RAASIs-free in cancer patients receiving ICIs treatment from inception to 1 November 2022 were retrieved by searching PubMed, Cochrane Library, Web of Science, Embase, and major conference proceedings. Studies in English reporting hazard ratios (HRs) with 95% confidence intervals (CIs) for overall survival (OS) and/or progression-free survival (PFS) were included. Statistical analyses were conducted using the software Stata 17.0.

**Results:**

A total of 12 studies containing 11739 patients were included, comprising ~4861 patients in the RAASIs-used and ICIs-treated group and ~6878 patients in RAASIs-free and ICIs-treated group. The pooled HR was 0.85 (95%CI, 0.75–0.96; *P* = 0.009) for OS and 0.91 (95%CI, 0.76–1.09; *P* = 0.296) for PFS, indicating a positive effect of RAASIs concomitant with ICIs on cancer patients. This effect was observed especially in patients with urothelial carcinoma (HR, 0.53; 95%CI, 0.31-0.89; *P* = 0.018) and renal cell carcinoma (HR, 0.56; 95%CI, 0.37-0.84; *P* = 0.005) on OS.

**Conclusion:**

Concomitant use of RAASIs and ICIs enhanced the efficacy of ICIs and this combination regimen was associated with significantly improved OS and a trend towards better PFS. RAASIs can be considered as adjuvant drugs when hypertensive patients receive ICIs treatment. Our results provide an evidence-based reference for the rational use of the RAASIs and ICIs combination therapy to improve the efficacy of ICIs in clinical practice.

**Systematic review registration:**

https://www.crd.york.ac.uk/prospero/, identifier CRD42022372636; https://inplasy.com/, identifier INPLASY2022110136.

## Introduction

Malignant tumors are associated with increased morbidity, high mortality, and heavy financial burden (1, 2). Currently, immune checkpoint inhibitors (ICIs) blocking programmed cell death-1 (PD-1), programmed cell death ligand-1 (PD-L1), or cytotoxic T lymphocyte-associated antigen 4 (CTLA-4), are promising therapeutic agents against multiple types of solid tumors (3–5). However, a large percentage of cancer patients still cannot benefit from ICIs due to the modest response rate or lacking of initial response (6, 7). Therefore, novel strategies to enhance the efficacy of ICIs are urgent clinical demands.

In the last decades, the renin-angiotensin-aldosterone system (RAAS) has been found to be linked to malignancies (8). Considerable preclinical studies have indicated that RAAS contributes to the formation of tumor immunosuppressive microenvironment (TME). Mechanistically, RAAS promotes the TME by upregulating the expression of PD-L1, producing immunosuppressive chemokines CC motif chemokine ligand 5 (CCL5), and enhancing the immunosuppressive activities of tumor-associated macrophages (TAMs), myeloid-deprived suppressive cells (MDSCs), and cancer-associated fibroblasts (CAFs) (9–11). The local RAAS in the TME stimulates angiogenesis by promoting the secretion of vascular endothelial growth factor (VEGF) (8). Additionally, RAAS can also induce cell proliferation and metastasis and drives the production of TAMs through angiotensin-converting enzyme/angiotensin II/angiotensin II type I receptor axis (8, 12, 13). Subsequently, an increasing number of studies demonstrate that renin-angiotensin-aldosterone system inhibitors (RAASIs) have immunomodulatory effects and can improve prognoses (14–17). Thus, the strategy of RAASIs, mainly including angiotensin-converting enzyme inhibitors (ACEI) and angiotensin receptor blockers (ARB), in combination with ICIs to enhance the therapeutic efficacy of inhibited ICIs was proposed (18, 19).

Hypertension is the most frequent comorbidity among cancer patients and one of the most common side effects of anticancer therapy (20, 21). Therefore, anticancer therapy concomitant with antihypertensive drugs is quite common and such circumstance will become more and more prevalent, especially with the aging of society. Among the antihypertensive drugs, RAASIs are frequently prescribed to patients with hypertension (22). Clinical studies have shown that RAASIs combined with chemo-radiotherapy are associated with better prognoses in several cancer types (23–25). But whether the concomitant use of RAASIs will augment the therapeutic efficacy of ICIs in a wide range of malignancies remains unclear. Given the necessity of finding novel strategies for enhancing the efficacy of tumor immunotherapy, we investigated the effect of RAASIs on prognoses in cancer patients receiving ICIs treatment and provided an evidence-based reference for the rational use of RAASIs and ICIs combination therapy in clinical practice.

## Methods

### Protocol and guideline

Registration of the full protocol was completed on dedicated websites (https://www.crd.york.ac.uk/prospero/ and https://inplasy.com/) as CRD42022372636 and INPLASY2022110136. The whole process of this study followed the statement of the Preferred Reporting Items for Systematic Reviews and Meta-Analyses (PRISMA) 2020 checklist (26).

### Search strategy

Studies evaluating the prognoses of RAASIs-used versus RAASIs-free in cancer patients receiving ICIs treatment from inception to 1 November 2022 were retrieved by searching PubMed, Cochrane Library, Web of Science, Embase, and major conference proceedings. Studies in English reporting hazard ratios (HRs) with 95% confidence intervals (CIs) for overall survival (OS) and/or progression-free survival (PFS) were included. The search queries were built with Mesh terms and free texts (**Supplementary Table 1**). The following broad terms were used to build the search queries: ‘immune checkpoint inhibitor’, ‘angiotensin-converting enzyme inhibitor’, ‘angiotensin receptor blocker’, ‘renin-angiotensin-aldosterone system’, and ‘cancer’.

### Selection criteria

All available studies in English evaluating survival outcomes in cancer patients treated with ICIs and RAASIs versus ICIs without RAASIs were included. Preclinical studies, case reports, and HR data not available studies were excluded. Studies with the largest sample sizes or the most up-to-date data were included when overlapping studies existed. The outcomes of interest were defined as HRs with 95%CIs for OS and PFS.

### Study selection, data extraction, and quality assessment

Two investigators (BL and XG) selected the literature according to the selection criteria independently. Any disagreements were discussed with another reviewer (YW) to reach a consensus. Information from included studies was abstracted by one investigator (BL) and checked by another reviewer (XG). Data from the multivariate analysis model was prior to adoption when both univariate and multivariate analysis model data were available. The extracted information was listed as follows: first author’s name, publication time and type, type of study, number and percentage of patients receiving RAASIs, sample size, type and stage of cancer, type of RAASIs, the regimen of ICIs, the time window of RAASIs use, analysis model, and HRs with 95%CIs for OS and PFS. Quality assessment was conducted using the modified Newcastle–Ottawa scale (NOS) (27). NOS scores of seven to nine were defined as high methodological quality, while of five or six were moderate quality and of four or less were low quality.

### Statistical analysis

All statistical analyses were calculated using the software Stata 17.0. HRs with corresponding 95%CIs were synthesized to yield pooled results. Heterogeneity among the included studies was assessed by the Cochrane *Q* test and *I^2^* value. *I^2^* > 50% and *P* < 0.1 for the *Q* test were considered significant heterogeneity and a random effect model was adopted. Otherwise, a fixed effect model was used. Subgroup and meta-regression analyses were performed to identify the potential heterogeneity contributors among the included studies. A funnel plot with Egger’s regression test was constructed to evaluate publication bias. Sensitivity analyses were conducted to examine the stability of the outcomes by the leaving-one-out approach. A two-tailed *P* < 0.05 was defined as statistical significance.

## Results

### Study selection

**Figure 1:**
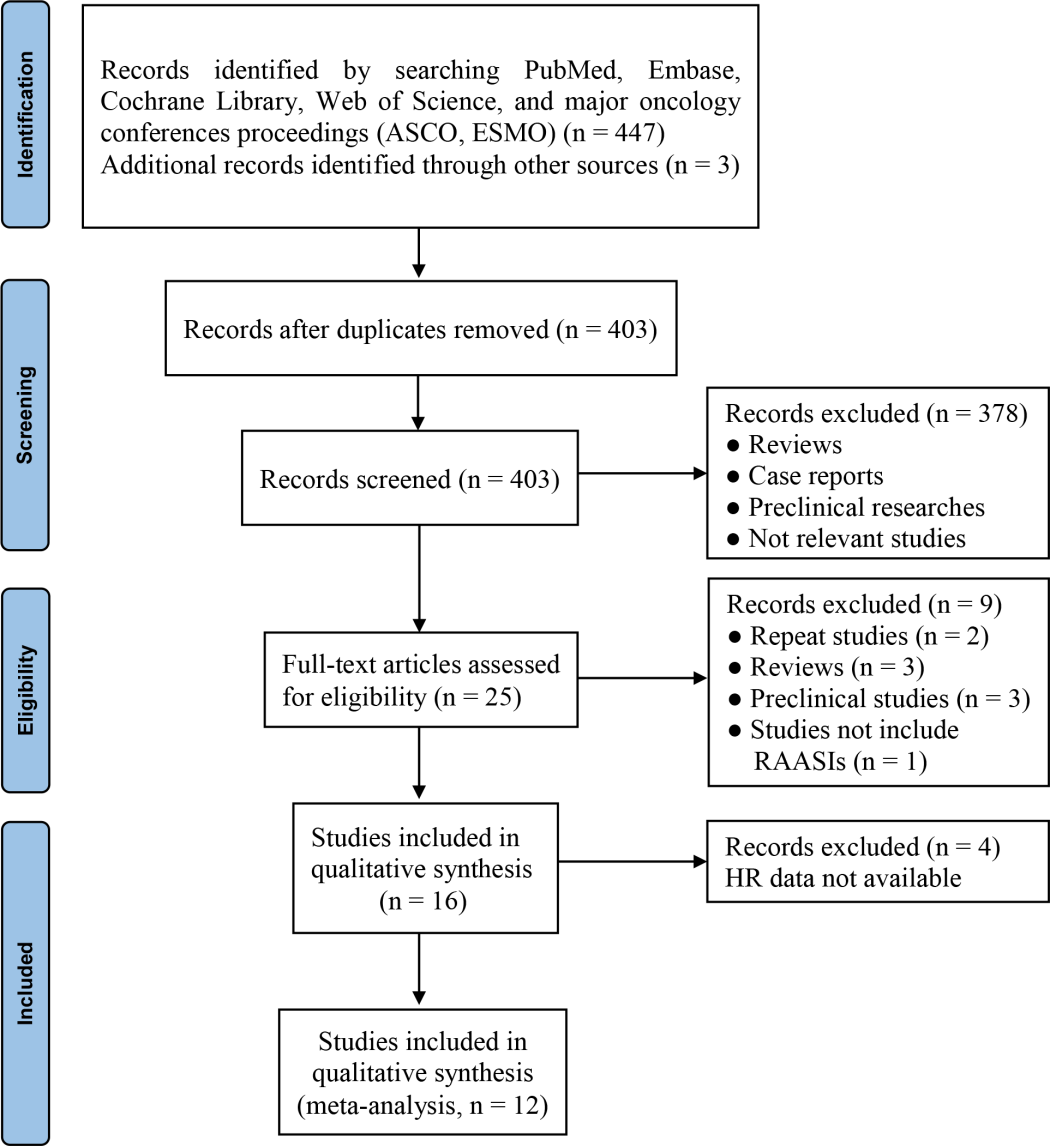
PRISMA flow diagram of study selection. ASCO, American Society of Clinical Oncology; ESMO, European Society for Medical Oncology; HR, hazard ratio; RAASIs, renin-angiotensin-aldosterone system inhibitors.

Initially, 450 records were identified from different sources. Finally, 12 studies (28–39) were included after duplicates removal, study selection, and eligibility assessment (**Figure 1**). Of the included studies, ten were peer-reviewed articles (28–30, 32–38) and two were meeting abstracts (31, 39).

### Characteristics and quality assessment

A total of 11739 (range, 80–5910) patients were enrolled, including 11739 patients analyzed for OS and 4827 patients for PFS. The publication types of these 12 studies were retrospective studies and integrated *post hoc* analyses. There were ~4861 RAASI-used patients and ~6878 RAASI-free patients. The prevalence of RAASIs use was observed ranging from 12.40% to 57.97%. Multiple cancer types were reported in six studies (30, 32, 33, 37–39). The regimen of ICIs included anti-PD-L1 monoclonal antibody (mAb) monotherapy, anti-CTLA-4 mAb monotherapy, anti-PD-1 or anti-PD-L1 mAb (anti-PD-1/L1), and anti-PD-1/L1 with or without anti-CTLA-4 mAb (anti-PD-1/L1 ± anti-CTLA-4). The time window of RAASIs use included simultaneous use (0, 30/NR) and baseline (**Supplementary Figure 1**). All studies were graded as high or moderate methodological quality (**Supplementary Table 2**). The characteristics and quality assessment results of included studies are shown in **Table 1**.

**Table 1:** Characteristics and quality assessment results of included studies.

Studies and Pub Date	Publication type	Type of study	Country/Region	No. of RAASIs use/No. of Patients (%)	Cancer type	Stage	ICIs used	RAASIs used	Time window of RAASIs use	Analysis model	HR for OS (95% CI)	HR for PFS (95% CI)	NOS
Jain et al 2021 (28)	Article	RP	USA	33/178 (18.5)	UC	Metastatic	Anti-PD-(L)1	ACEI/ARB	(0, NR)	MVA	0.52 (0.29-0.93)	NR	7
				22/101 (21.8)	UC	Metastatic	Anti-PD-(L)1	ACEI/ARB	(0, NR)	MVA	0.57 (0.17-1.96)	NR	
Medjebar et al 2020 (29)	Article	RP	Europe	22/178 (12.4)	NSCLC	Locally advanced/ Metastatic	Anti-PD-(L)1	ACEI	(0, NR)	MVA	1.96 (1.09–3.51)	1.89 (1.14–3.16)	6
Kostine et al 2021 (30)	Article	RP	Europe	203/635 (32.0)	Multiple	Advanced	Anti-PD-(L)1±Anti-CTLA-4	ACEI/ARB	Baseline	UVA	0.98 (0.79-1.21)	1.01 (0.83-1.23)	7
				NR/293	Melanoma	Advanced	Anti-PD-(L)1±Anti-CTLA-4	ACEI/ARB	Baseline	UVA	1.24 (0.91-1.69)	0.94 (0.70-1.26)	
				NR/150	NSCLC	Advanced	Anti-PD-(L)1±Anti-CTLA-4	ACEI/ARB	Baseline	UVA	0.78 (0.51-1.19)	1.04 (0.69-1.57)	
				NR/83	RCC	Advanced	Anti-PD-(L)1±Anti-CTLA-4	ACEI/ARB	Baseline	UVA	0.74 (0.40-1.36)	1.24 (0.74-2.08)	
Pereira et al 2021 (31)	Abstract	RP	Europe	35/127 (27.6)	NSCLC	NR	Anti-PD-(L)1±Anti-CTLA-4		(0, NR)	NR			6
								ARB	(0, NR)	NR	0.44 (0.19-1.02)	0.40 (0.17-0.93)	
								ACEI	(0, NR)	NR	0.75 (0.39-1.42)	0.87 (0.46-1.65)	
Buti et al 2021 (32)	Article	RP	Europe	66/217 (30.6)	Multiple	Advanced Metastatic	Anti-PD-(L)1±Anti-CTLA-4	ACEI	Baseline	UVA	0.69 (0.48-1.01)	NR	**7**
Kichenadasse et al 2021 (33)	Article	*post hoc* analysis	Multiple	604/2539 (23.8)	Multiple	Advanced Metastatic	Atezolizumab	ACEI/ARB or Both	(0, NR)	MVA	0.92 (0.79-1.07)	0.95 (0.84-1.08)	8
Failing et al 2016 (34)	Article	RP	USA	11/80 (13.8)	Melanoma	Advanced	Ipilimumab	ACEI/ARB	(0, NR)	MVA	0.41 (0.10-1.71)	0.67 (0.33-1.36)	9
Nuzzo et al 2022 (35)	Article	RP	USA	89/229 (38.9)	RCC	Metastatic	Anti-PD-(L)1±Anti-CTLA-4	ACEI/ARB	(0, 30)	MVA			6
				30/100 (30.0)	RCC	Metastatic	Anti-PD-(L)1±Anti-CTLA-4	ACEI/ARB	(0, 30)	MVA	0.35 (0.17-0.70)	NR	
				59/129 (45.7)	RCC	Metastatic	Anti-PD-(L)1±Anti-CTLA-4	ACEI/ARB	(0, 30)	MVA	0.60 (0.34-1.06)	NR	
Tozuka et al 2021 (36)	Article	RP	Japan	37/256 (14.5)	NSCLC	NR	Anti-PD-(L)1	ACEI/ARB	(0, NR)	UVA	0.71 (0.45-1.11)	0.59 (0.40-0.88)	8
Cortellini et al 2020 (37)	Article	RP	Europe	313/1012 (30.9)	Multiple	Advanced	Anti-PD-(L)1	ACEI/ARB	Baseline	MVA	0.91 (0.74-1.11)	0.94 (0.79-1.12)	**7**
Drobni et al 2022 (38)	Article	RP	USA	3426/5910 (57.97)	Multiple	NR	Anti-PD-(L)1±Anti-CTLA-4	ACEI/ARB	(0, NR)	MVA	0.90 (0.84-0.98)	NR	7
				555/913 (60.8)	Melanoma	NR	Anti-PD-(L)1±Anti-CTLA-4	ACEI/ARB	(0, NR)	UVA	1.02 (0.81-1.27)	NR	
Cortellini et al 2020 (39)	Abstract	RP	Europe	NR/277	Multiple	Advanced	Anti-PD-(L)1	ACEI/ARB	Baseline	NR	1.14 (0.81-1.62)	NR	**7**

ICIs, immune checkpoint inhibitors; RAASIs, renin-angiotensin-aldosterone system inhibitors; HR, hazard ratio; OS, overall survival; PFS, progression-free survival; RP, retrospective study design; UC, urothelial carcinoma; NSCLC, non-small cell lung cancer; RCC, renal cell carcinoma; NR, not reported; PD-1, programmed cell death-1; PD-L1, programmed cell death 1 ligand; CTLA-4, cytotoxic T lymphocyte-associated antigen 4; ACEI, angiotensin-converting enzyme inhibitors; ARB, angiotensin receptor blockers; MVA, multivariate analysis; UVA, univariate analysis.

### Pooled OS and PFS

Jain et al. (28) and Nuzzo et al. (35) provided prognostic outcomes for two independent cohorts and Pereira et al. (31) reported prognostic outcomes for two divided cohorts categorized by RAASIs type rather than the whole population. Hence, we integrated the outcomes of these three studies separately. Finally, a total of 15 and 8 cohorts reported HR data for OS and PFS, respectively. Three cohorts (28, 35, 38) were observed a significantly positive effect on OS and two cohorts (31, 36) on PFS for the concomitant use of RAASIs and ICIs. Eleven cohorts (28, 30–37, 39) for OS and seven cohorts (30, 31, 33, 34, 37) for PFS were observed no significant impact on prognoses in cancer patients treated with ICIs and RAASIs versus ICIs without RAASIs. Of note, one cohort (29) was observed a significantly negative impact on both OS and PFS in patients receiving ICIs concomitant with RAASIs. The above contradictory results confirmed the necessity of our study once again. Consequently, the method of integrating different studies through meta-analysis would be feasible to resolve this issue.

**Figure 2:**
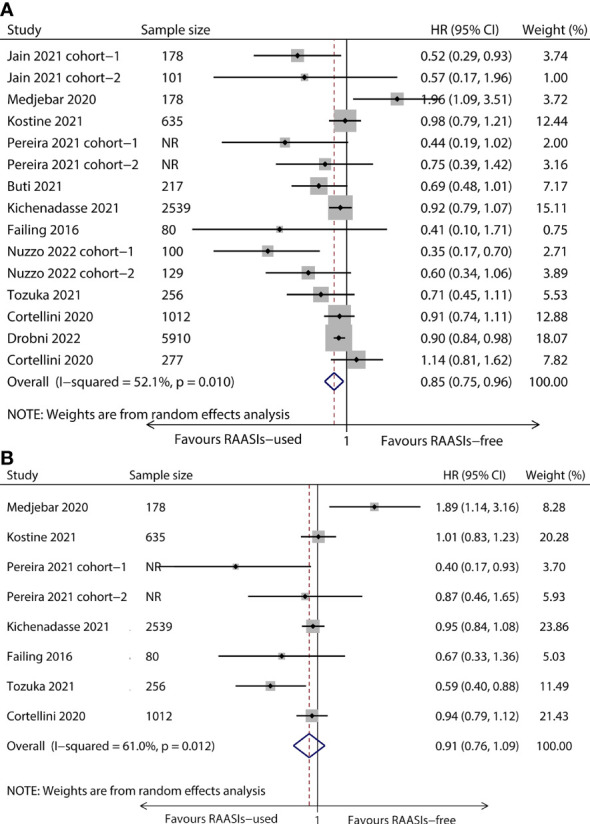
Forest plot of OS and PFS comparing RAASIs-used and RAASIs-free patients treated with ICIs. Pooled HR for OS **(A)** and PFS **(B)**. OS, overall survival; PFS, progression-free survival.

The heterogeneity among included studies was significant (*I^2 =^* 52.1%, *P* = 0.010 for OS and *I^2 =^* 61.0%, *P* = 0.012 for PFS) and a random effect model was adopted to calculate pooled results. Meta-analysis showed that the pooled HR was 0.85 (95%CI, 0.75–0.96; *P* = 0.009) for OS (**Figure 2A**) and 0.91 (95%CI, 0.76–1.09; *P* = 0.296) for PFS (**Figure 2B**), which meant a significantly improved OS and a tendency for better PFS were found in the population receiving ICIs concomitant with RAASIs.

### Subgroup analysis

To explore potential factors contributing to heterogeneity among the included studies, subgroup and meta-regression analyses were conducted by various factors, including cancer type, the time window of RAASIs use, RAASIs type, the regimen of ICIs, geographical region, and analysis model.

**Figure 3:**
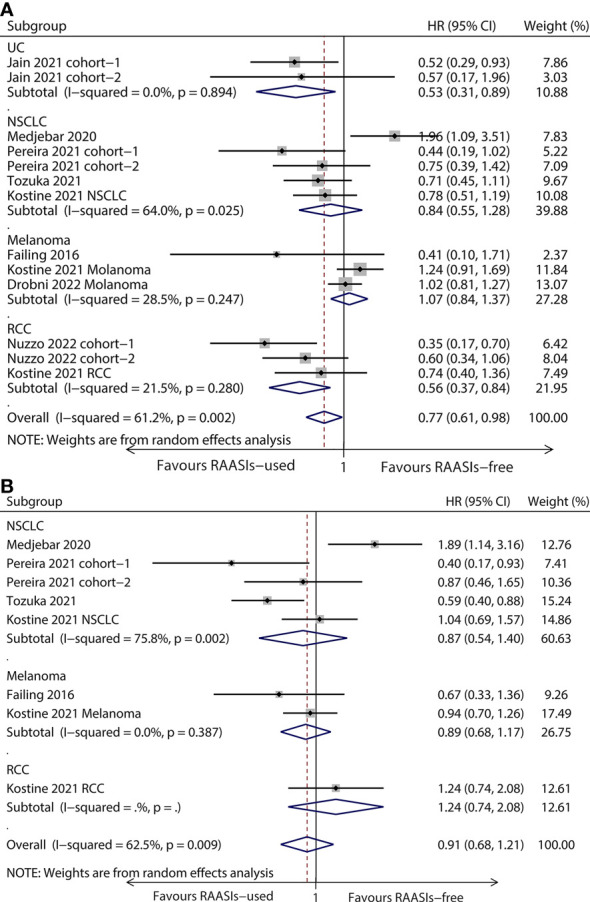
Forest plots of subgroup analysis by cancer type. Results for OS **(A)** and PFS **(B)**. OS, overall survival; PFS, progression-free survival; HR, hazard ratio; UC, urothelial carcinoma; NSCLC, non-small cell lung cancer; RCC, renal cell carcinoma.

The population was divided into four subgroups based on cancer type, comprising urothelial carcinoma (UC), non-small cell lung cancer (NSCLC), renal cell carcinoma (RCC), and melanoma. Subgroup analyses showed that there were longer OS in UC (HR, 0.53; 95%CI, 0.31-0.89; *P* = 0.018) and RCC (HR, 0.56; 95%CI, 0.37-0.84, *P* = 0.005) subgroup, while there was no statistical significance for PFS (**Figure 3**). Both OS and PFS were observed no statistical significance in NSCLC and melanoma subgroup (**Figure 3**).

Subgroup analyses were also performed according to the period during which RAASIs were used, including simultaneous use (0, 30/NR) and baseline. Subgroup analyses showed that there was longer OS in simultaneous use (HR, 0.77; 95%CI, 0.64-0.93; *P* = 0.041) subgroup, while there was no statistical significance for PFS (**Supplementary Figure 2**). Both OS and PFS were observed no statistical significance in baseline subgroup (**Supplementary Figure 2**).

Subgroups categorized by the regimen of ICIs included anti-PD-L1 mAb monotherapy, anti-CTLA-4 mAb monotherapy, anti-PD-1/L1 mAb, and Anti-PD-1/L1 ± Anti-CTLA-4 mAb. Subgroup analyses showed that there was longer OS in Anti-PD-1/L1 ± Anti-CTLA-4 mAb (HR, 0.76; 95%CI, 0.62-0.93; *P* = 0.008) subgroup, while there was no statistical significance for PFS (**Supplementary Figure 3**). Both OS and PFS were observed no statistical significance in anti-PD-L1 mAb monotherapy, anti-CTLA-4 mAb monotherapy, and anti-PD-1/L1 subgroup (**Supplementary Figure 3**).

According to the resident region of patients, including USA, Europe, and Japan, patients were classified into three subgroups. Subgroup analyses showed that there was longer OS in USA (HR, 0.60; 95%CI, 0.40-0.88; *P* = 0.009) subgroup, while there was no statistical significance for PFS (**Supplementary Figure 4**). There was a longer PFS in Japan (HR, 0.59; 95%CI, 0.40-0.88; *P* = 0.009) subgroup, while there was no statistical significance for OS (**Supplementary Figure 4**).

Cohorts were divided into two subgroups according to the analysis model, comprising multivariate and univariate analysis. Subgroup analyses showed that both OS and PFS were observed no statistical significance in two subgroups (**Supplementary Figure 5**).

### Meta-regression analysis

Subgroup analyses indicated that subgroup factors including cancer type, the time window of RAASIs use, the regimen of ICIs, the residential region of patients, and the analysis model were not the factors contributing to the statistically significant heterogeneity. RAASIs type was also considered in meta-regression besides the previously mentioned factors, but no factors were detected as the causes of statistically significant heterogeneity (**Supplementary Table 3**).

### Publication bias and sensitivity analysis

In the funnel plot for OS, there were some asymmetries in the distribution (**Supplementary Figure 6A**). However, as indicated by Egger’s regression test, no significant publication bias was detected (*P* = 0.103) (**Supplementary Figure 6B**). Sensitivity analyses showed that both the pooled HRs for OS and PFS were not significantly affected by individual cohorts, indicating results were robust (**Supplementary Figure 7**).

## Discussion

To our knowledge, this study is the first to summarize the current evidence evaluating the prognostic effect of RAASIs in cancer patients receiving ICIs treatment through meta-analysis. Compared with previous studies, this meta-analysis with the inclusion of multiple cancer types and more than 10,000 patients makes the results more reliable and convincing.

The included studies comprising multiple cancer types enabled us to investigate whether RAASIs combined with ICIs will have the same effect on different malignancies. Thus, we performed subgroup analyses to better understand the efficacy of this combination therapy for specific cancer types. Of the common cancer types, we observed that ICIs concomitant with RAASIs improved the survival outcomes in patients with UC and RCC, but not patients with NSCLC and melanoma, indicating there may be a cancer-type-specific effect for this combination therapy. Consequently, the underlying mechanism needs to be further clarified and more studies on specific cancer types are needed.

Subgroup analyses categorized by the time window of RAASIs use showed that only the simultaneous use of RAASIs and ICIs therapy lengthened OS. This may be explained by the characteristics of the patients. Compared with patients receiving simultaneous use of RAASIs, patients receiving baseline RAASIs tended to be older and had more comorbidities. Besides, these patients may concomitantly use multiple drugs, such as antibiotics, glucocorticoids, and proton pump inhibitors, which are considered to be associated with negative survival outcomes (40–42). Additionally, drug-drug interaction may exist in baseline co-medications, which may be antagonistic to this combination therapy. However, whether the time window of RAASIs use and the sequence of drug administration will impact the effect of this combination regimen on prognoses in cancer patients needs to be further explored.

The results of subgroup analyses suggested that there may be regional differences in this combination therapy. Patients from USA showed a better OS, which is consistent with the pooled HR for OS. On the contrary, patients from Japan had a longer PFS. These may be attributed to the differences in sensitivity of different races to RAASIs. It is reported that the activity of RAAS of African Americans is different from that of white people (43). Besides, the effect of RAASIs on African Americans is less effective than on whites (44, 45).

RAASIs type and dosage may affect the efficacy of this combination therapy. But the association between RAASIs type and dosage with survival outcomes is still under debate. These analyses were not performed due to the lack of desirable data. According to Drobni et al, RAASIs type did not affect the efficacy of this combination therapy for OS; however, a dose-dependent effect was observed (38). Consequently, studies to investigate the impact of RAASIs type and dosage on the therapeutic efficacy of this combination strategy in cancer patients are warranted.

Importantly, although this combination therapy may improve the efficacy of ICIs, safety is also an unavoidable issue. Two large-sample studies reported that RAASIs in combination with ICIs did not significantly increase immune-related adverse events (irAEs) (33, 38). Therefore, this combination strategy may be an effective and safe therapy, which not only improves survival outcomes but also does not develop significant differences in the incidence of irAEs. Our studies may shed some light on the rational use of this combination strategy.

Of note, our studies showed that there was just a trend of better PFS, instead of a significantly improved PFS. One possible explanation for the observation is that PFS is usually judged based on image data, the results are affected by the evaluation interval, and the results may be inconsistent with the OS benefit. In this study, we included 12 studies but only 7 studies reported PFS. Consequently, we recommend that future studies with similar themes could provide as many prognostic outcomes as possible.

Additionally, RAASIs are safe, low-cost, and commonly prescribed antihypertensive agents (22). Therefore, the small investment in adopting RAASIs as adjuvant antihypertensive drugs when patients with onco-hypertension receive ICIs therapy may have a significant impact on public health and gain great benefits. Given that cancer treatment causes heavy financial burden to the national finance and the individual (46), the combination of RAASIs and ICIs may decrease medical expenditure and save medical resources thus contributing to relieving the financial burden of cancer treatment.

There were certain limitations in our study. This meta-analysis was based on clinical studies, which were not able to provide mechanistic insights for the anti-tumor effect of this combination regimen. Besides, some results of subgroup analyses should be interpreted with caution because of the undesired sample size. Due to the limited data for RAASIs type and stage of cancer, these subgroup analyses were not performed. Moreover, the included studies were retrospective and *post hoc* analyses with the nature of inevitable confounding factors in observational studies, the actual effect of the combination of RAASIs and ICIs on prognoses in cancer patients would preferably be confirmed through the well-designed randomized clinical trial in future.

## Conclusion

This large-scale study revealed that ICIs concomitant with RAASIs enhanced the efficacy of ICIs and this combination regimen was associated with significantly improved OS and a trend of better PFS. RAASIs can be considered as adjuvant drugs when hypertensive patients receive ICIs treatment. Our results provide an evidence-based reference for the rational use of this combination therapy to improve the efficacy of ICIs in clinical practice.

## Data availability statement

The original contributions presented in the study are included in the article/**Supplementary Material**. Further inquiries can be directed to the corresponding authors.

## Author contributions

JS and HH designed the study and drafted the manuscript. BL and XG performed data acquisition, quality assessment, and statistical analyses. LC, YW, and YY provided funding support and revised and approved the final manuscript. All authors contributed to the article and approved the submitted version.
