# Supplementary material for: Effect of renin-angiotensin-aldosterone system inhibitors on survival outcomes in cancer patients treated with immune checkpoint inhibitors: a systematic review and meta-analysis
Source: Front Immunol. 2023 Apr 19;14:1155104. doi: 10.3389/fimmu.2023.1155104 (PMC10154532; doi:10.3389/fimmu.2023.1155104)

**Supplementary Material**

**Effect of renin-angiotensin-aldosterone system inhibitors on survival outcomes in cancer patients treated with immune checkpoint inhibitors: a systematic review and meta-analysis**

**Running Title:** Combination of RAAIs and ICIs augment the cancer immunotherapy

**Authors:** Jinhai Shen^1, 2†^, Hui Hou^1, 2†^, Bowen Liang^1, 2^, Xiao Guo^1, 2^, Li Chen^4^, Yong Yang^1, 2, 3*^, Yun Wang^1,2*^

**^*^Corresponding authors:**

1. Yun Wang, Center for New Drug Safety Evaluation and Research, State Key Laboratory of Natural Medicines, China Pharmaceutical University, Nanjing, Jiangsu 211198, PR China. Email: [wangyun@cpu.edu.cn](mailto:wangyun@cpu.edu.cn)
2. Yong Yang, Center for New Drug Safety Evaluation and Research, State Key Laboratory of Natural Medicines, China Pharmaceutical University, Nanjing, Jiangsu 211198, PR China; School of Pharmacy, Xuzhou Medical University, Xuzhou, Jiangsu, 221004, PR China. Email: yy@cpu.edu.cn

**Supplementary Table 1.** Details of searching strategy.

**Supplementary Table 2.** Quality assessment of included studies through the modified Newcastle-Ottawa Quality Assessment Scale (NOS).

**Supplementary Table 3.** Meta-regression analysis of factors contributing to the heterogeneity among the included studies.

**Supplementary Figure 1.** Schematic illustration of the time window of RAASIs use.

**Supplementary Figure 2.** Forest plots for overall and progression-free survival of subgroups by the time window of RAASIs use

**Supplementary Figure 3.** Forest plots for overall and progression-free survival of subgroups by the regimen of ICIs.

**Supplementary Figure 4.** Forest plots for overall and progression-free survival of subgroups by geographical region.

**Supplementary Figure 5.** Forest plots for overall and progression-free survival of subgroups by analysis model.

**Supplementary Figure 6.** Publication bias for included studies on overall survival using funnel plot and Egger’s regression test.

**Supplementary Figure 7.** Sensitivity analyses for included studies on overall and progression-free survival examined by leaving-one-out approach.

**Supplementary Table 1** Details of searching strategy.

| **Database** | **Search strategy** |
| --- | --- |
| ***PubMed*** | (("Immune Checkpoint Inhibitors"[Mesh] OR ‘Immune Checkpoint Blocker*’ OR PD-1 OR anti-PD-1 OR ‘PD-1 Inhibitor*’ OR ‘Programmed Cell Death Protein 1 Inhibitor*’ OR PD-L1 OR anti-PD-L1 OR ‘PD-L1 Inhibitor*’ OR ‘Programmed Death Ligand 1 Inhibitor*’ OR ‘PD-1 Inhibitor*’‘Programmed Death Ligand 1 Inhibitor*’ OR CTLA-4 OR anti-CTLA-4 OR ‘CTLA-4 Inhibitor*’ OR ‘Cytotoxic T Lymphocyte-Associated Protein 4 Inhibitor*’)) AND (((("Angiotensin-Converting Enzyme Inhibitors"[Mesh] OR ‘Angiotensin-Converting Enzyme Antagonist*’ OR ‘ACE Inhibitor*’ OR ‘Angiotensin I-Converting Enzyme Inhibitor*’ OR ‘Angiotensin-Converting Enzyme Inhibitor*’)) OR (("Receptors, Angiotensin"[Mesh] OR ‘Angiotensin Receptor Blocker*’ OR ‘Angiotensin Receptor Antagonist*’ OR ‘Angiotensin II Receptor Antagonist*’ OR ‘Angiotensin II Receptor Blocker*’))) OR (("Renin-Angiotensin System"[Mesh] OR Renin-Angiotensin-Aldosterone System)))  **Search results: 151 items** |
| ***Cochrane Library*** | \| ID \| Search \| \| --- \| --- \| \| #1 \| MeSH descriptor: [Immune Checkpoint Inhibitors] explode all trees \| \| #2 \| PD-1 OR anti-PD-1 OR 'PD-1 Inhibitor*' OR 'Programmed Cell Death Protein 1 Inhibitor*' OR PD-L1 OR anti-PD-L1 OR 'PD-L1 Inhibitor*' OR 'Programmed Death Ligand 1 Inhibitor*' OR CTLA-4 OR anti-CTLA-4 OR 'CTLA-4 Inhibitor*' OR 'Cytotoxic T Lymphocyte-Associated Protein 4 Inhibitor*' \| \| #3 \| #1 OR #2 \| \| #4 \| MeSH descriptor: [Angiotensin-Converting Enzyme Inhibitors] explode all trees \| \| #5 \| MeSH descriptor: [Angiotensin Receptor Antagonists] explode all trees \| \| #6 \| MeSH descriptor: [Renin-Angiotensin System] explode all trees \| \| #7 \| #4 OR #5 OR #6 \| \| #8 \| ACEI OR 'ACE Inhibitor*' OR ARB OR 'Angiotensin Receptor Blocker*' OR 'Renin-Angiotensin system' OR 'Renin-Angiotensin-Aldosterone System' \| \| #9 \| #7 OR #8 \| \| #10 \| #3 AND #9 \|   **Search results: 11 items** |
| ***Web of Science*** | TS=( ‘Immune Checkpoint Inhibitor* OR ‘Immune Checkpoint Blocker*’ OR PD-1 OR anti-PD-1 OR ‘PD-1 Inhibitor*’ OR ‘Programmed Cell Death Protein 1 Inhibitor*’ OR PD-L1 OR anti-PD-L1 OR ‘PD-L1 Inhibitor*’ OR ‘Programmed Death Ligand 1 Inhibitor*’ OR CTLA-4 OR anti-CTLA-4 OR ‘CTLA-4 Inhibitor*’ OR ‘Cytotoxic T Lymphocyte-Associated Protein 4 Inhibitor*’) AND TS=(‘Angiotensin Converting Enzyme Inhibitor*’ OR ‘Angiotensin-Converting Enzyme Antagonist*’ OR ‘ACE Inhibitor*’ OR ‘Angiotensin I-Converting Enzyme Inhibitor*’ OR ‘Angiotensin-Converting Enzyme Inhibitor*’ OR ‘Angiotensin Receptor Blocker*’ OR ‘Angiotensin Receptor Antagonist*’ OR ‘Angiotensin II Receptor Antagonist*’ OR ‘Angiotensin II Receptor Blocker*’) AND TS=(Neoplasm* OR Tumor* OR Cancer* OR Malignanc*)  **Search results: 94 items** |
| ***Embase*** | ('immune checkpoint inhibitors'/exp OR 'Immune Checkpoint Blocker*' OR PD-1 OR anti-PD-1 OR 'PD-1 Inhibitor*' OR 'Programmed Cell Death Protein 1 Inhibitor*' OR PD-L1 OR anti-PD-L1 OR 'PD-L1 Inhibitor*' OR 'PD-L1 Inhibitor*' OR 'Programmed Death-Ligand 1 Inhibitor*' OR CTLA-4 OR anti-CTLA-4 OR 'CTLA-4 Inhibitor*' OR 'Cytotoxic T Lymphocyte-Associated Protein 4 Inhibitor*') AND ('Angiotensin Converting Enzyme Inhibitors'/exp OR 'Angiotensin-Converting Enzyme Antagonist*' OR 'Angiotensin-Converting Enzyme Inhibitor*' OR 'Angiotensin I-Converting Enzyme Inhibitor*' OR 'Angiotensin Converting Enzyme Inhibitor*' OR 'ACE Inhibitor*' OR 'Angiotensin I Converting Enzyme Inhibitor*' OR 'Antagonists, Angiotensin Receptor'/exp OR 'Angiotensin Receptor Blocker*' OR 'Angiotensin Receptor Antagonist*' OR 'Angiotensin II Receptor Antagonist*' OR 'Angiotensin II Receptor Blocker*') AND ('Neoplasm'/exp OR 'Tumor*' OR 'Neoplasia*' OR 'Cancer*' OR 'Malignant Neoplasm*' OR 'Malignanc*')  **Search results: 221 items** |
| ***ASCO, ESMO*** | (("Immune Checkpoint Inhibitor*") OR ("Immune Checkpoint Blocker*") OR ("PD L1 Inhibitor*") OR ("Programmed Death Ligand 1 Inhibitor*") OR ("CTLA 4 Inhibitor*") OR ("Cytotoxic T Lymphocyte-Associated Protein 4 Inhibitor*") OR ("PD 1 Inhibitor*") OR ("Programmed Cell Death Protein 1 Inhibitor*")) AND (("angiotensin-converting enzyme inhibitor*") OR ("angiotensin receptor blocker*") OR (“renin-angiotensin system”)) |

**Supplementary Table 2** Quality assessment of included studies through the modified Newcastle-Ottawa Quality Assessment Scale (NOS).

| Studies | Selection | | | | Compatibility | Assessment | | | Total stars | Score |
| --- | --- | --- | --- | --- | --- | --- | --- | --- | --- | --- |
|  | Representativeness of the exposed cohort | Selection of the non-exposed cohort | Ascertainment of exposure | Demonstration that outcome of interest was not present at the start of study | Comparability of cohorts on the basis of the design or analysis* | Assessment of outcome | Was follow-up long enough for outcomes to occur | Adequacy of follow up of cohorts |  |  |
| Jain et al 2021 | ★ | ★ | ★ | ★ | ★ (1, 3, 4, 9) | ★ | ★ | - | 7★ | 7 |
| Medjebar  et al 2020 | ★ | ★ | ★ | ★ | ★ (1, 2, 4, 9) | ★ | - | - | 6★ | 6 |
| Kostine et al 2021 | ★ | ★ | ★ | ★ | ★ (3, 4, 7, 9) | ★ | - | ★ | 7★ | 7 |
| Pereira et al 2021 | ★ | ★ | ★ | ★ | - (1, 3, 9) | ★ | ★ | - | 6★ | 6 |
| Buti et al 2021 | ★ | ★ | ★ | ★ | ★ (2, 3, 4, 6, 9) | ★ | - | ★ | 7★ | 7 |
| Kichenadasse  et al 2021 | ★ | ★ | ★ | ★ | ★ (3, 4, 6, 8, 9) | ★ | ★ | ★ | 8★ | 8 |
| Failing et al 2016 | ★ | ★ | ★ | ★ | ★★ (1, 2, 3, 4, 6, 8, 9) | ★ | ★ | ★ | 9★ | 9 |
| Nuzzo et al 2022 | ★ | ★ | ★ | ★ | -(1, 2, 9) | ★ | ★ | - | 6★ | 6 |
| Tozuka et al 2021 | ★ | ★ | ★ | ★ | ★★ (1, 3, 4, 6, 7, 8, 9) | ★ | - | ★ | 8★ | 8 |
| Cortellini et al 2020 | ★ | ★ | ★ | ★ | ★ (2, 3, 4, 9) | ★ | - | ★ | 7★ | 7 |
| Drobni et al 2022 | ★ | ★ | ★ | ★ | ★ (3, 4, 7, 8, 9) | ★ | ★ | - | 7★ | 7 |
| Cortellini et al 2020 | ★ | ★ | ★ | ★ | ★ (2, 3, 4, 9) | ★ | - | ★ | 7★ | 7 |

*1. Cancer type, 2. stage, 3. time window of RAASIs use, 4. the regimen of ICIs, 5. treatment line of ICIs, 6. Eastern Cooperative Oncology Group Performance Status (ECOG PS), 7. age, 8. gender, and 9. race were considered. If seven to nine factors of two cohorts were comparable, two stars were assigned; if four to six factors of two cohorts were comparable, one star was assigned; otherwise, no star was assigned. Studies assigned with score of seven to nine were defined as high methodological quality, while of five or six were moderate quality and of four or less were low quality. The score is equal to the total number of stars.

**Supplementary Table 3** Meta-regression analysis of factors contributing to the heterogeneity among the included studies.

| **Factor** | **OS** | | | |
| --- | --- | --- | --- | --- |
|  | **SE** | ***t*** | **Coefficient (95%CI)** | ***P*** |
| **Cancer type** |  | | | |
| RCC | Reference | | | |
| UC | 0.42 | -0.09 | 0.96 (0.35, 2.59) | 0.931 |
| NSCLC | 0.47 | 1.36 | 1.53 (0.76, 3.08) | 0.206 |
| Melanoma | 0.62 | 1.96 | 1.89 (0.31, 3.95) | 0.082 |
| **Time window of RAASIs use** |  | | | |
| Baseline | Reference | | | |
| Simultaneous use | 0.16 | -1.04 | 0.92 (0.66, 1.28) | 0.316 |
| **Region** |  | | | |
| Japan | Reference | | | |
| USA | 0.35 | -0.35 | 0.87 (0.36, 2.10) | 0.733 |
| Europe | 0.50 | 0.70 | 1.31 (0.56, 3.06) | 0.496 |
| **RAASIs type** | 0.31 | 0.14 | 1.04 (0.53, 2.06) | 0.895 |
| **Regimen of ICIs** |  | | | |
| CTLA-4 | Reference | | | |
| PD-(L)1 | 1.86 | 0.97 | 2.24 (0.36, 13.89) | 0.352 |
| PD-(L)1**±**CTLA-4 | 1.44 | 0.67 | 1.74 (0.28, 10.73) | 0.516 |
| PD-L1 | 1.95 | 0.93 | 2.24 (0.33, 15.18) | 0.372 |
| **Analysis model** |  | | | |
| UVA | Reference | | | |
| MVA | 0.25 | -0.02 | 1.00 (0.57, 1.74) | 0.986 |

Abbreviations: OS, overall survival; SE, standard error; CI, confidence interval; ICIs, immune checkpoint inhibitors; RAASIs, renin-angiotensin-aldosterone system inhibitors; UC, urothelial carcinoma; NSCLC, non-small cell lung cancer; RCC, renal cell carcinoma; NR, not report; PD-1, programmed cell death-1; PD-L1, programmed cell death ligand 1; CTLA-4, cytotoxic T lymphocyte–associated antigen 4; ACEI, angiotensin-converting enzyme inhibitors; ARB, angiotensin receptor blockers; MVA, multivariate analysis; UVA, univariate analysis

**
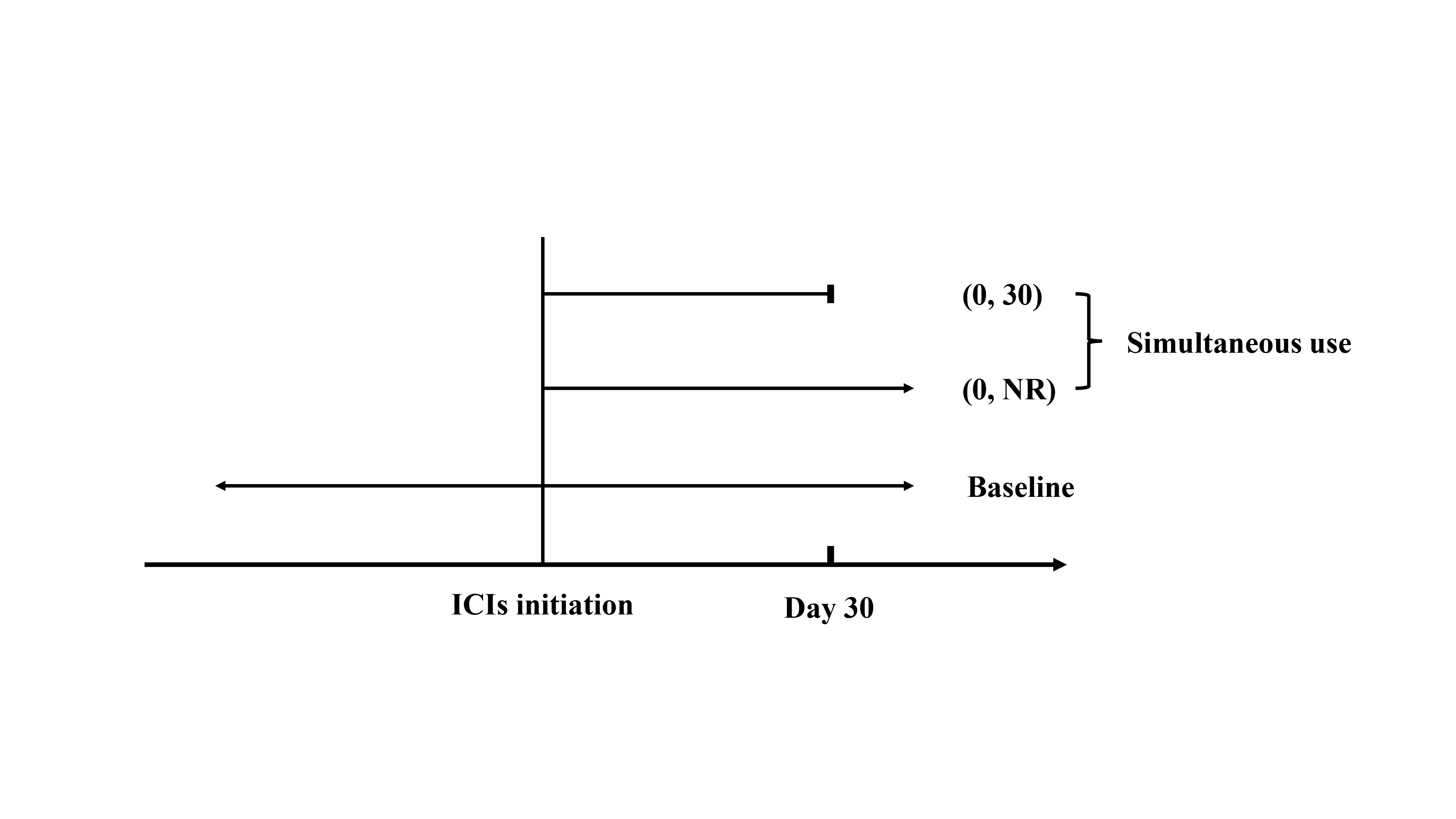
Supplementary Figure 1.** Schematic illustration of the time window of RAASIs use. Abbreviation: RAASIs, renin-angiotensin-aldosterone system inhibitors; ICIs, immune checkpoint inhibitors; NR, not reported.

**Supplementary Figure 2.** Forest plots for overall and progression-free survival of subgroups by the time window of RAASIs use. Results for OS **(A)** and PFS **(B)**. Abbreviation: OS, overall survival; PFS, progression-free survival; CI, confidence interval; RAASIs, renin-angiotensin-aldosterone system inhibitors; NR, not reported.


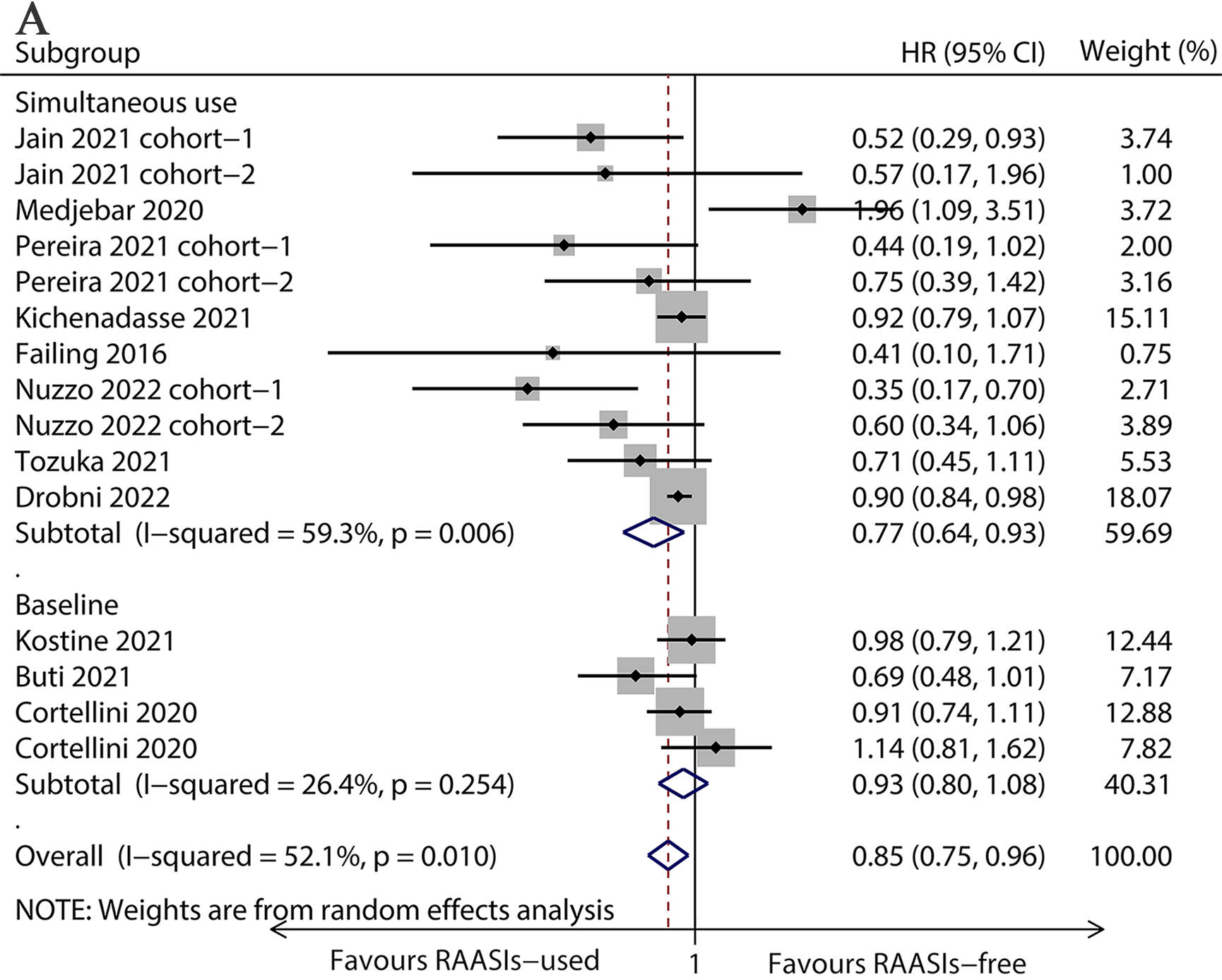

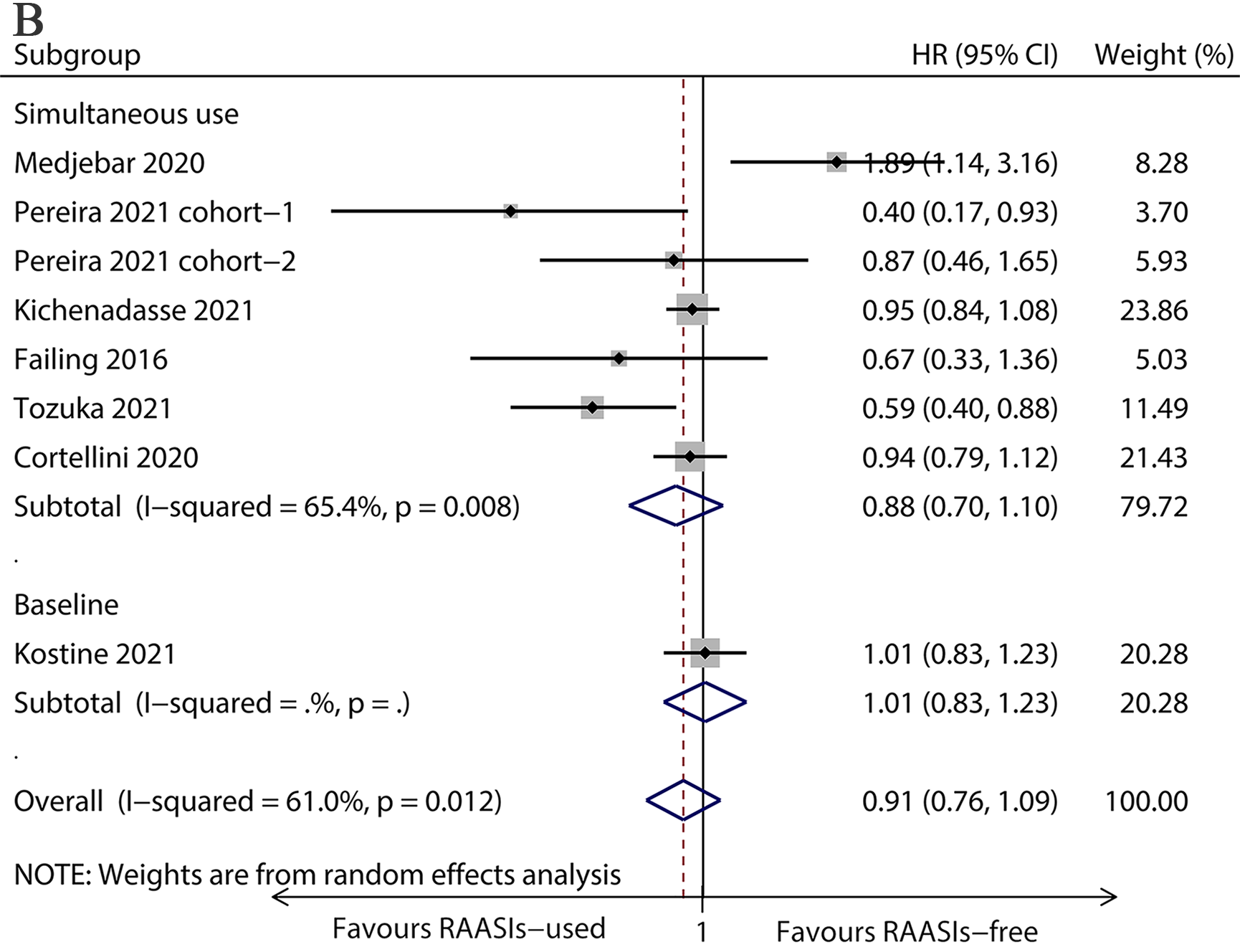


**Supplementary Figure 3.** Forest plots for overall and progression-free survival of subgroups by the regimen of ICIs. Results for OS **(A)** and PFS **(B)**. Abbreviation: OS, overall survival; PFS, progression-free survival; CI, confidence interval; PD-1, programmed cell death-1; PD-L1, programmed cell death ligand 1; CTLA-4, cytotoxic T lymphocyte–associated antigen 4.


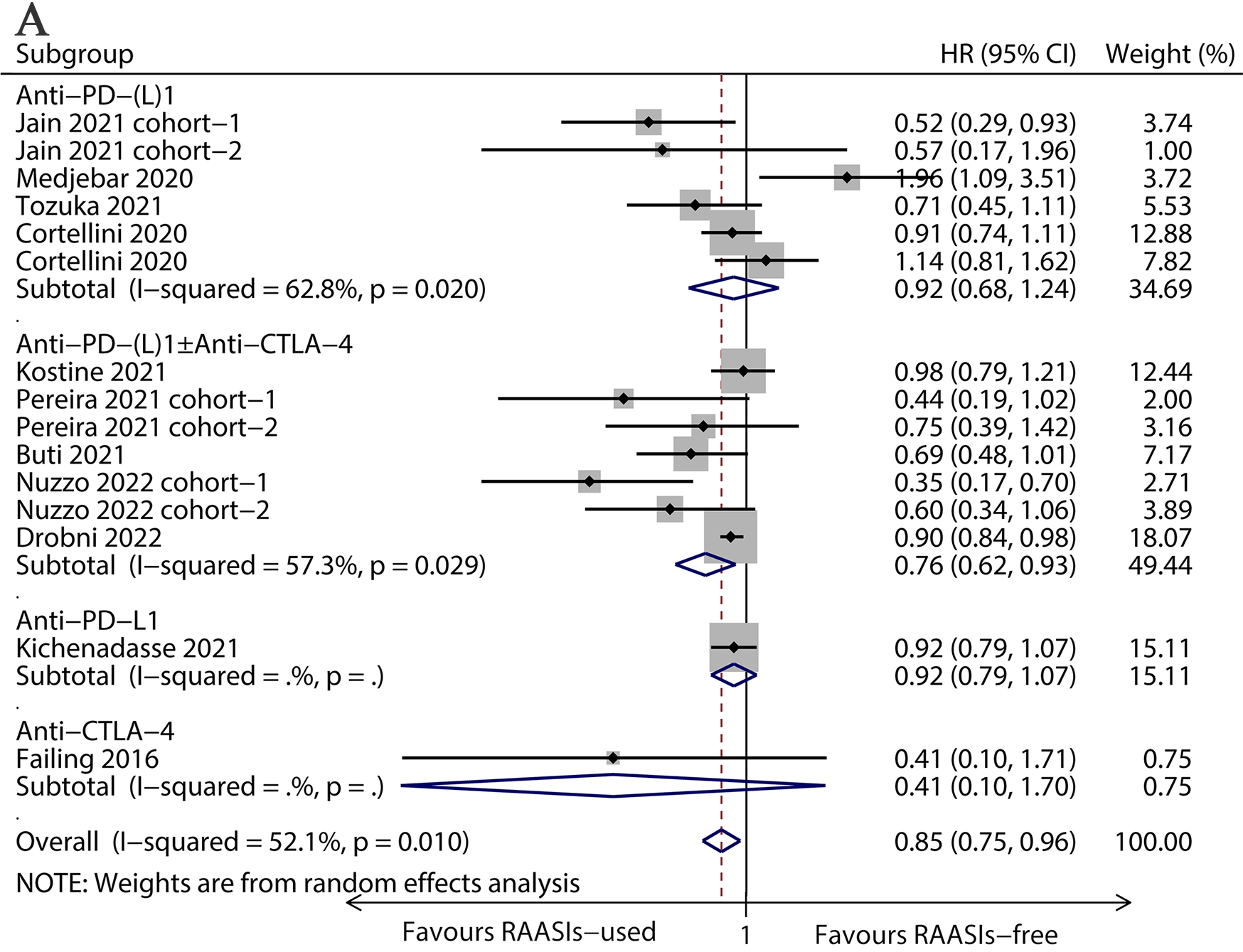

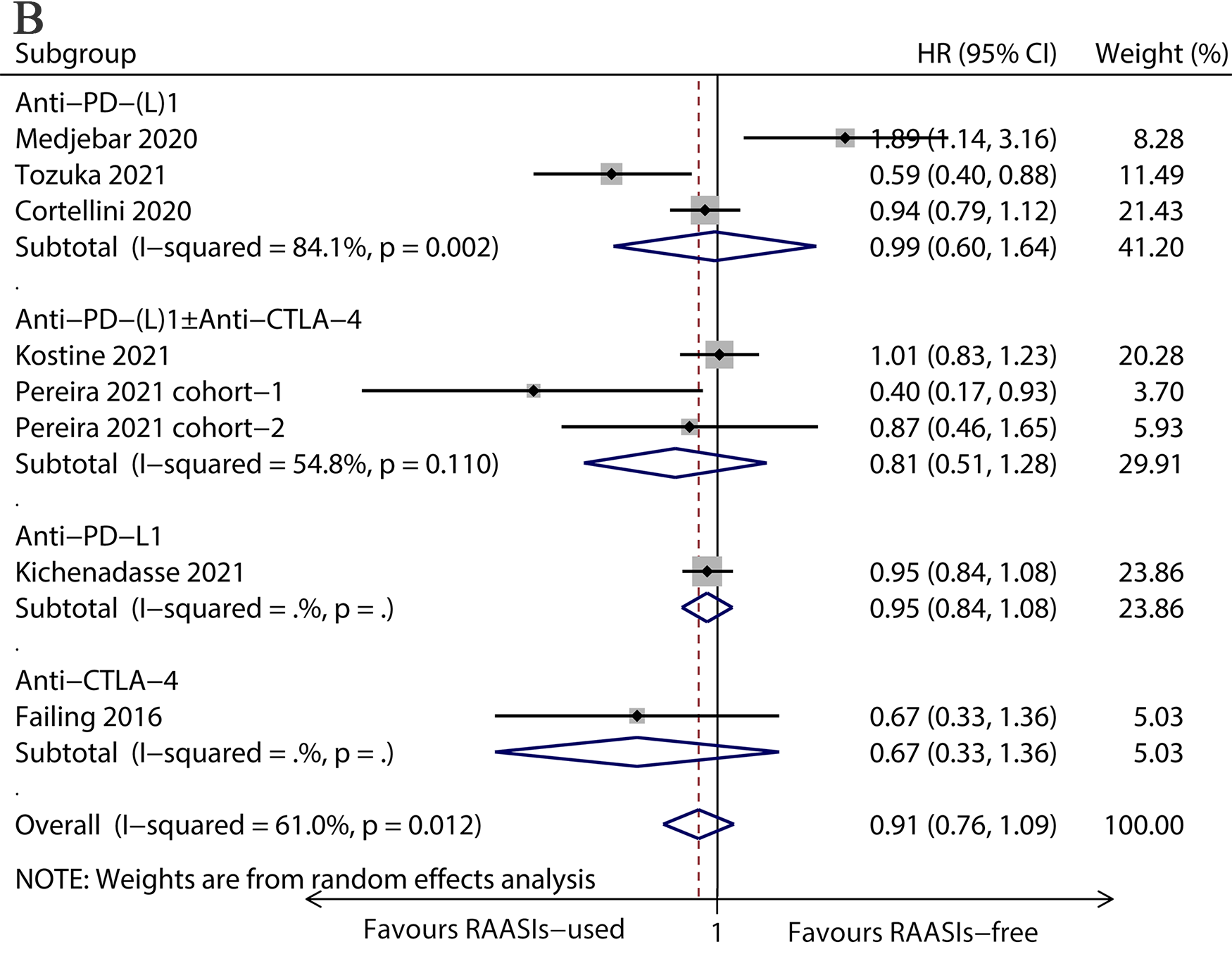


**Supplementary Figure 4.** Forest plots for overall and progression-free survival of subgroups by geographical region. Results for OS **(A)** and PFS **(B)**. Abbreviation: OS, overall survival; PFS, progression-free survival; CI, confidence interval.


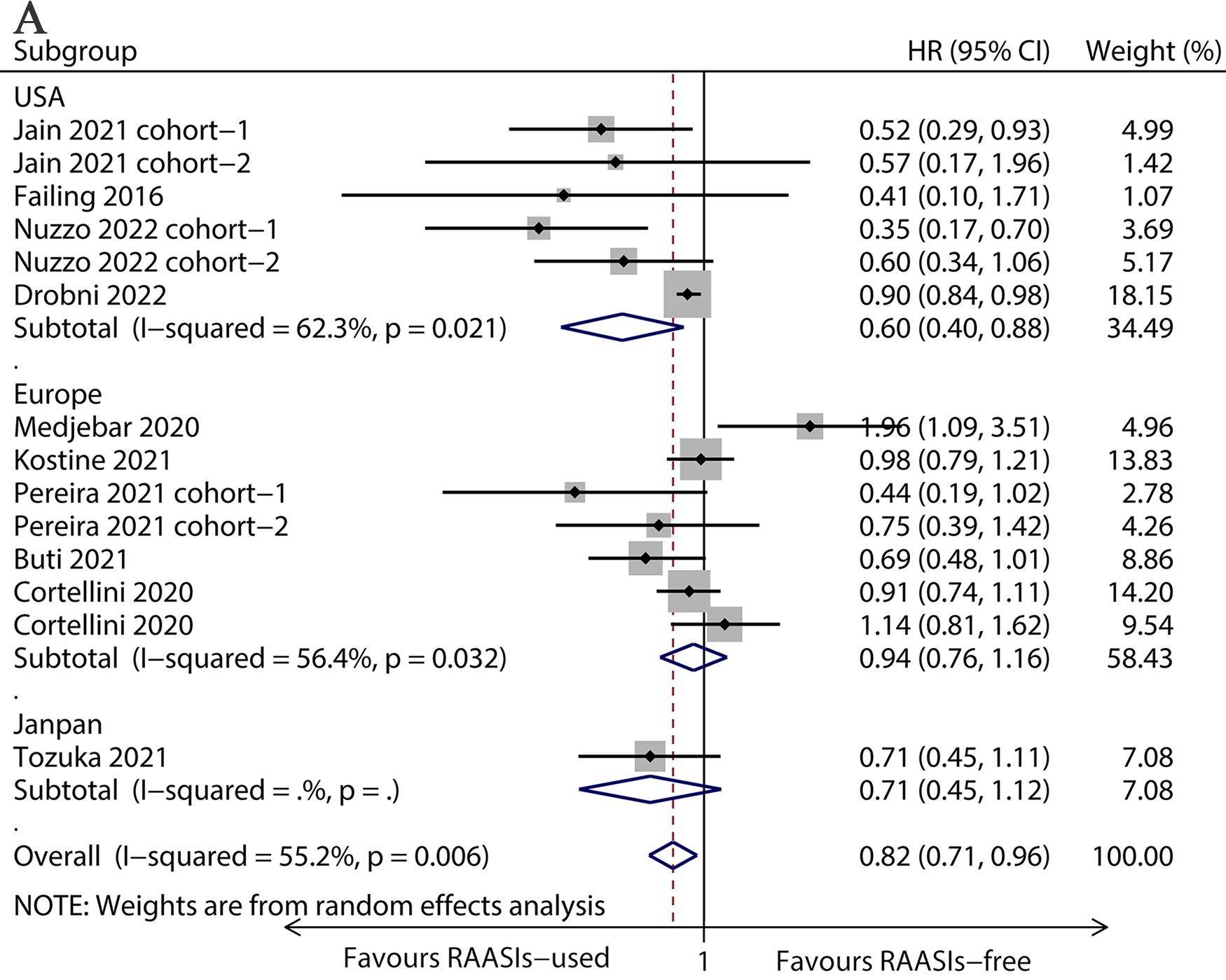

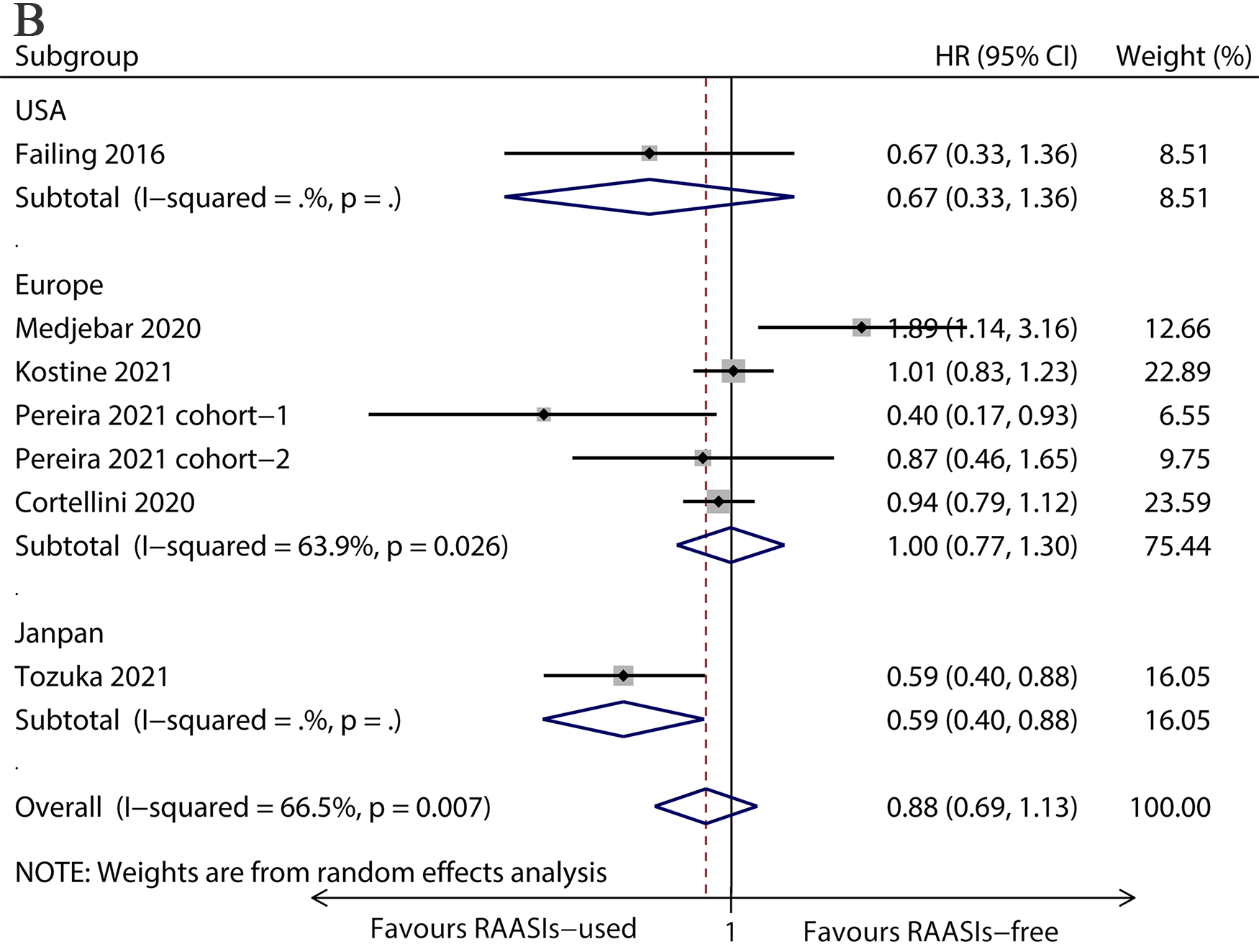


**Supplementary Figure 5.** Forest plots for overall and progression-free survival of subgroups by analysis model. Results for OS **(A)** and PFS **(B)**. Abbreviation: OS, overall survival; PFS, progression-free survival; CI, confidence interval; MVA, multivariate analysis; UVA, univariate analysis.


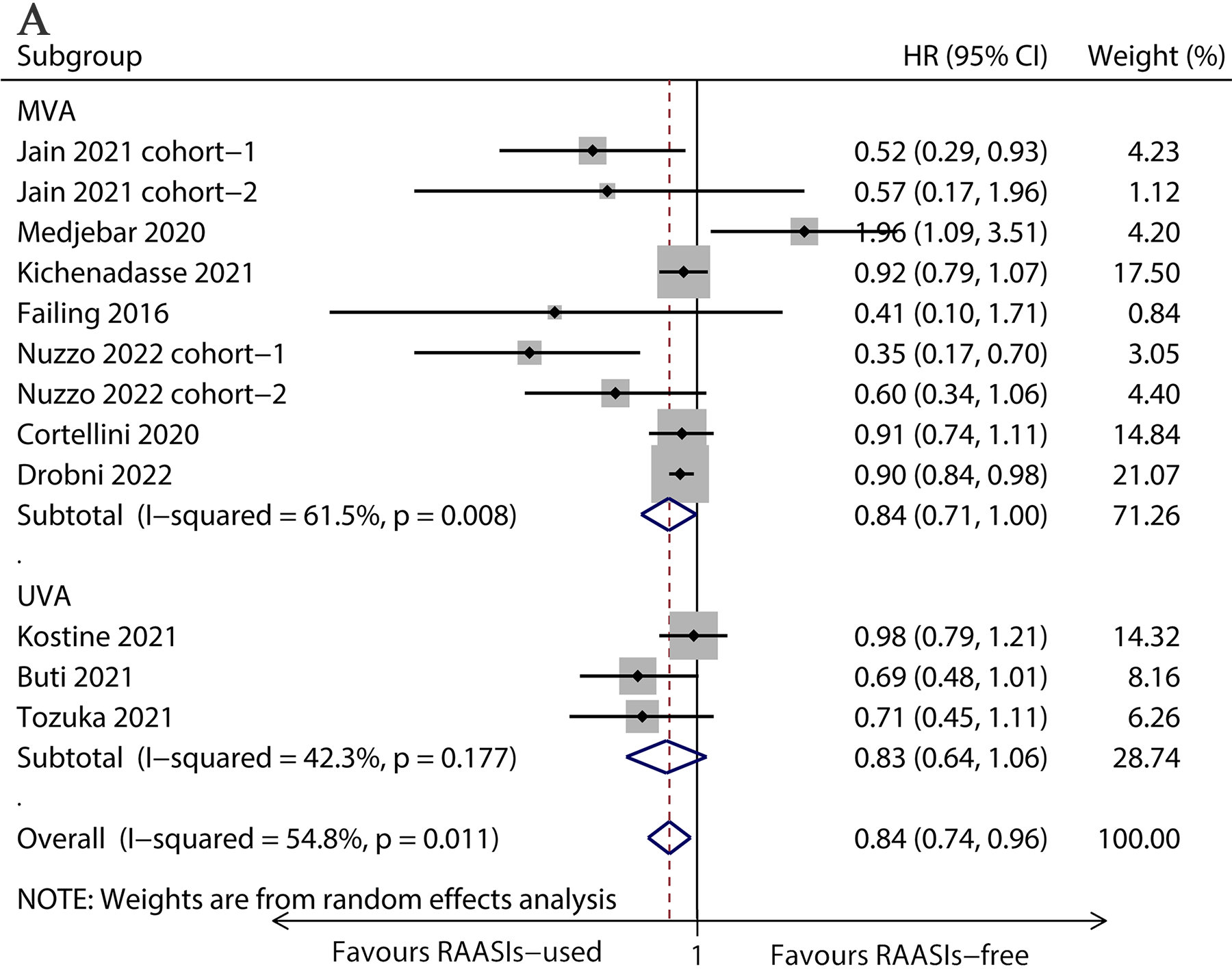

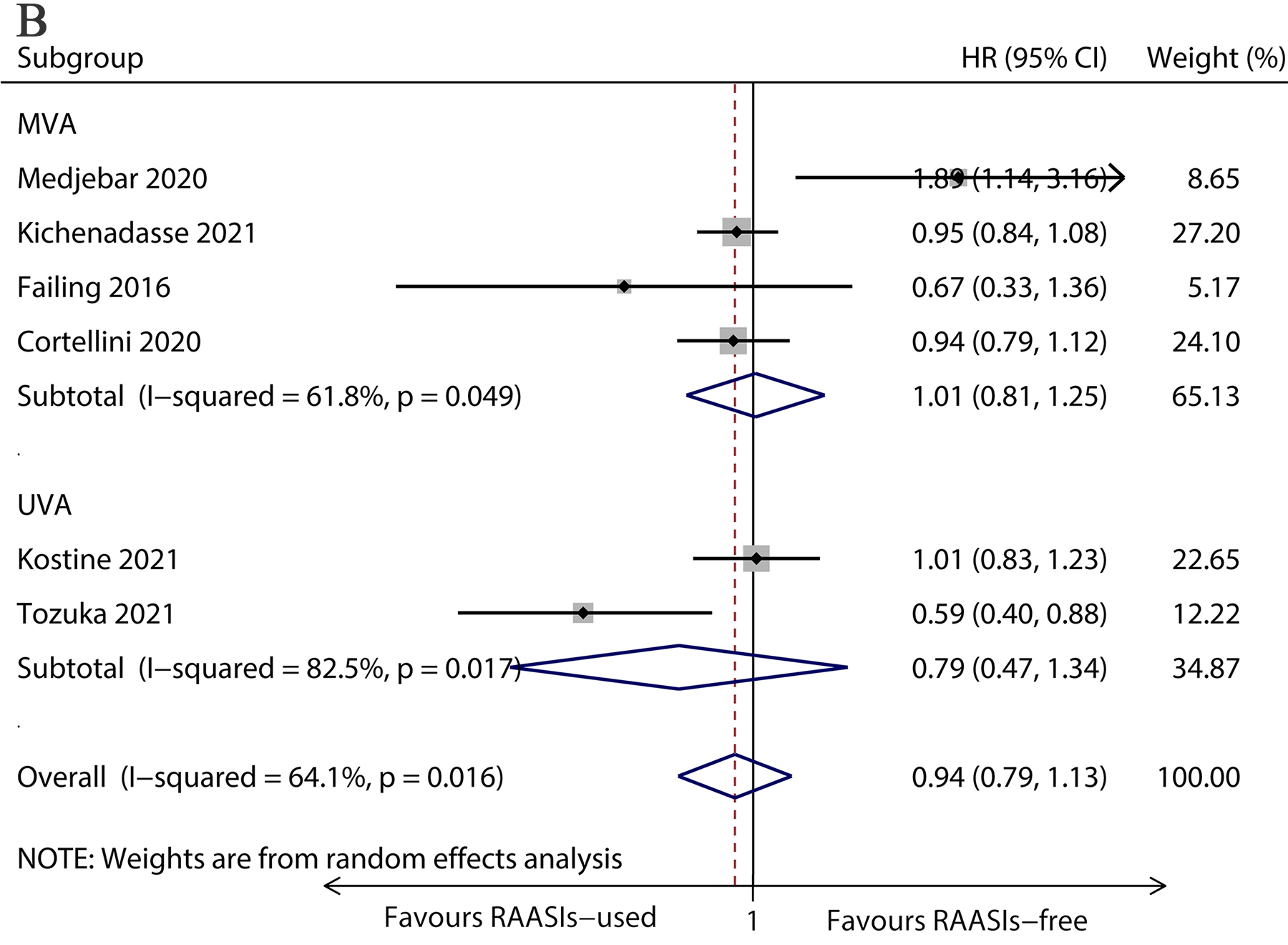


**Supplementary Figure 6.** Publication bias for included studies on overall survival using funnel plot and Egger’s regression test. Funnel plot **(A)** and Egger’s regression test **(B)** of publication bias for OS. Abbreviations: OS, overall survival; HR, hazard ratio; CI, confidence interval; SND, standardized.


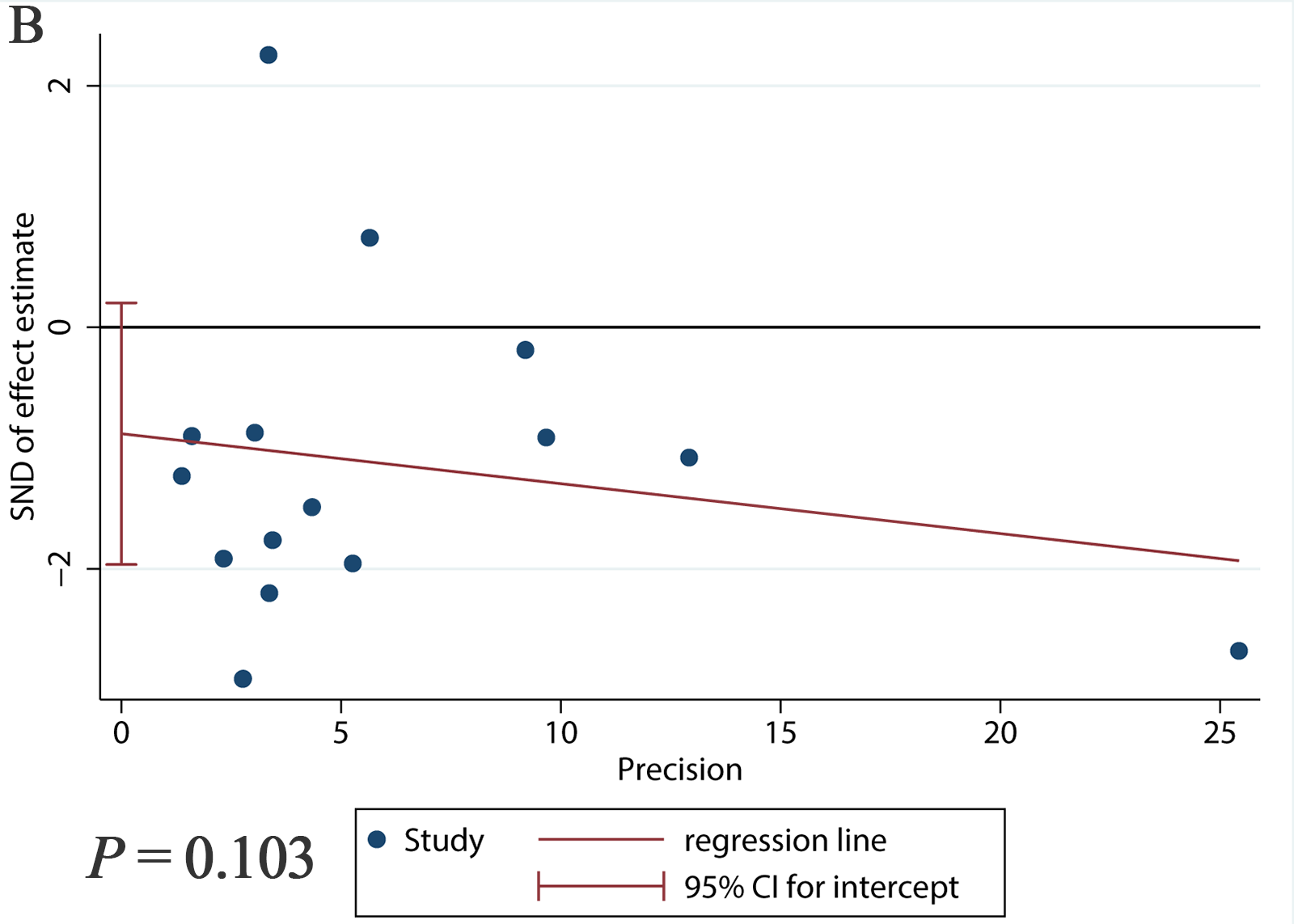

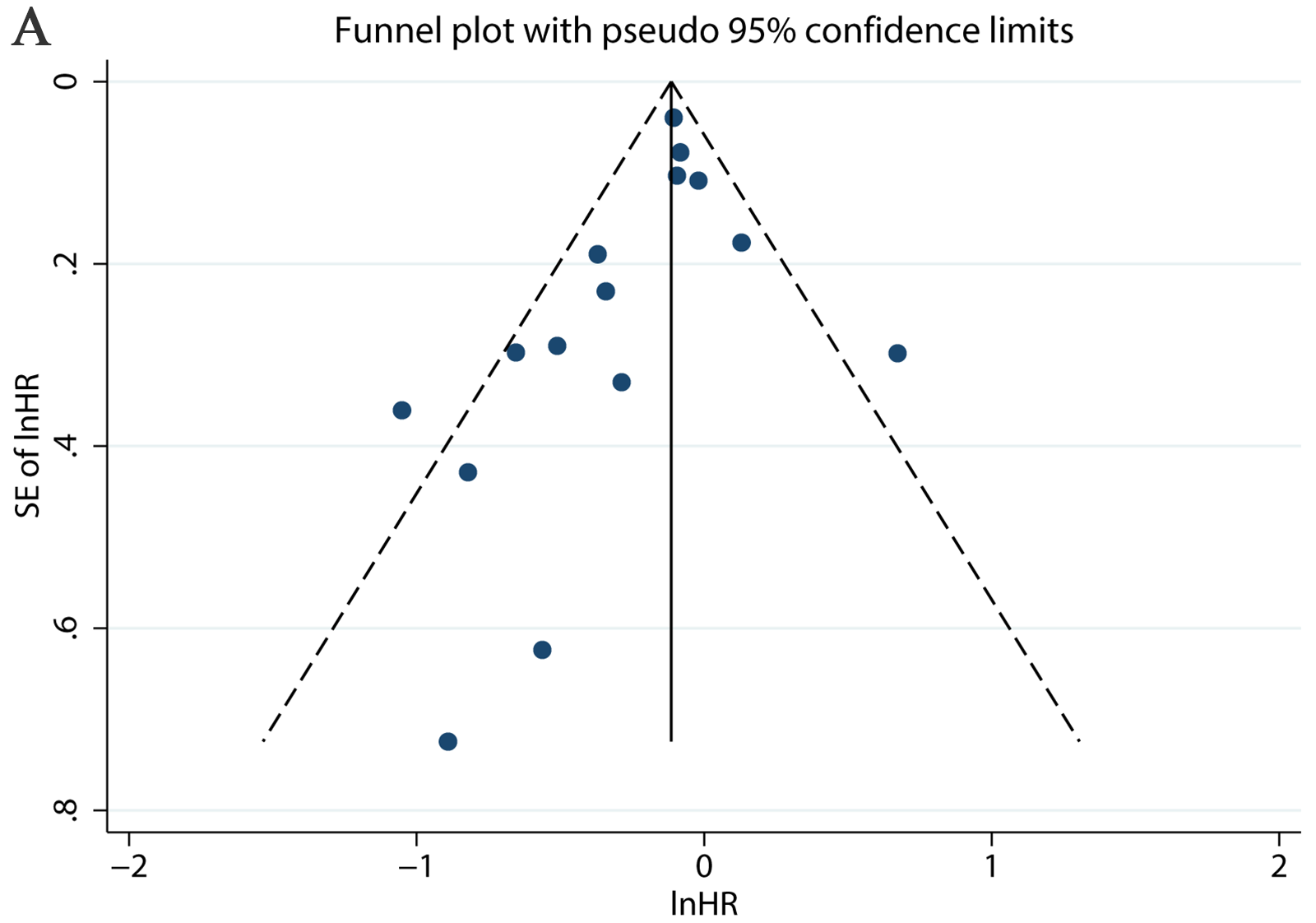


**Supplementary Figure 7.** Sensitivity analyses for included studies on overall and progression-free survival examined by leaving-one-out approach. Results for OS **(A)** and PFS **(B)**. Abbreviations: OS, overall survival; PFS, progression-free survival; CI, confidence interval.


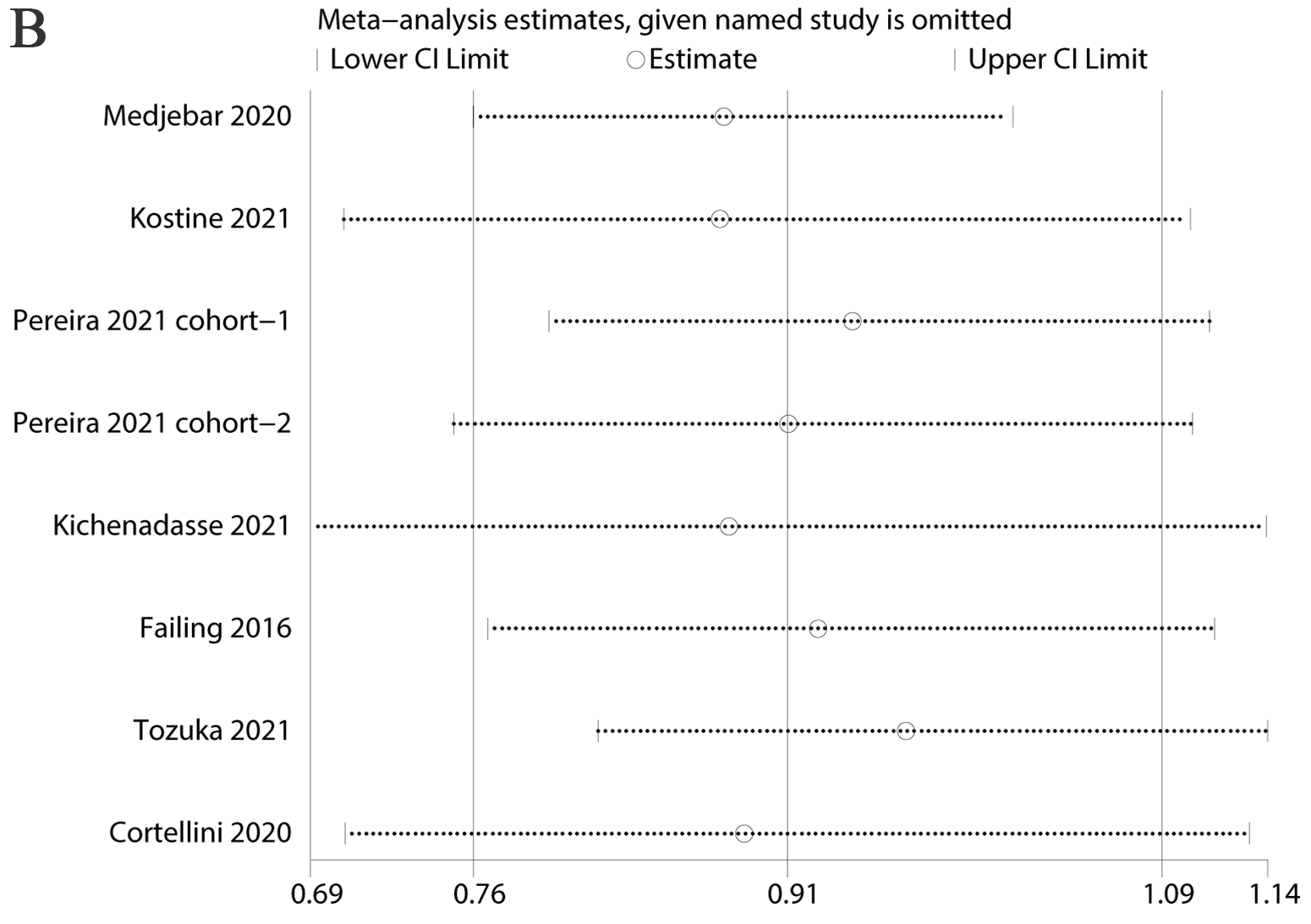

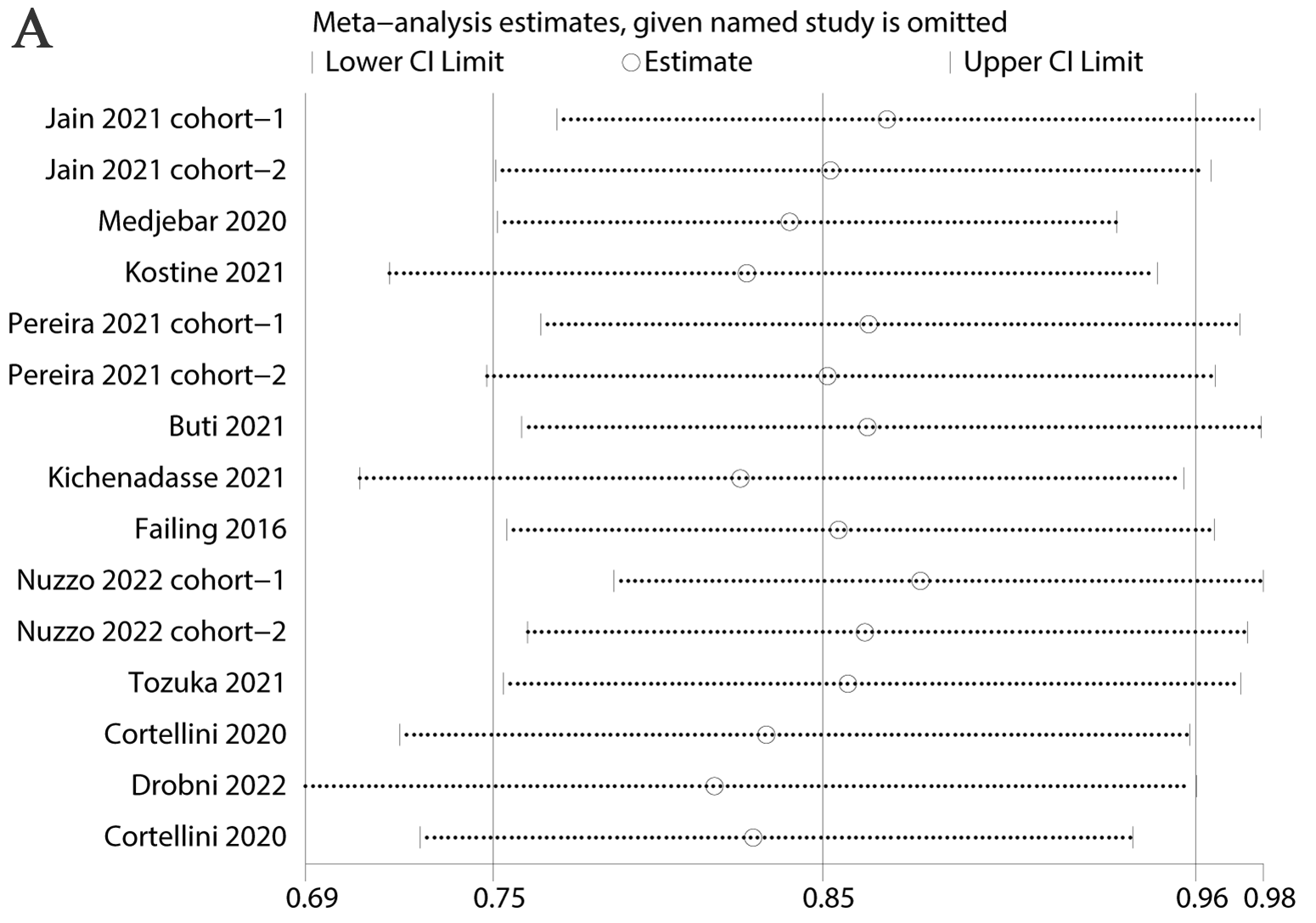

Supplement: Supplementary file 1 [file DataSheet_1.docx]
